# Extended-release of doxorubicin through green surface modification of gold nanoparticles: in vitro and in ovo assessment

**DOI:** 10.1186/s13065-022-00895-x

**Published:** 2022-12-06

**Authors:** Maryam Asariha, Seyed Hossein Kiaie, Sepideh Izadi, Faezeh H. Pirhayati, Mehdi Fouladi, Maryam Gholamhosseinpour

**Affiliations:** 1 Medical Biology Research Center, Health Technology Institute, Kermanshah University of Medical Sciences, Kermanshah, Iran; 2 Nano Drug Delivery Research Center, Health Technology Institute, Kermanshah University of Medical Sciences, Kermanshah, Iran; 3 Department of Pharmaceutics, Faculty of Pharmacy, Tabriz University of Medical Sciences, Tabriz, Iran; 4 Department of Immunology, Faculty of Medicine, Tabriz University of Medical Sciences, Tabriz, Iran; 5 Pharmaceutical Sciences Research Center, School of Pharmacy, Kermanshah University of Medical Sciences, Kermanshah, Iran

**Keywords:** Chondroitin sulfate, Gold nanoparticles, Chitosan, Doxorubicin, Cytotoxicity, Green synthesis, Drug delivery

## Abstract

In the present study, a green surface modification of gold nanoparticles (GNPs) using chondroitin sulfate (CHS) and chitosan (CS) to deliver an extended-release of doxorubicin (DOX) was proposed. Following synthesis of each step of unconjugated counterpart, including CHS-GNPs, DOX–CHS–GNP, and conjugated construct DOX–CHS–GNP-CS, physicochemical properties of the nanoparticles (NPs) were characterized by FT-IR, DLS, and TEM analyses, and the release of DOX was determined by using UV–Vis spectrometry. Then, NPs were effectively taken up by MDA-MB-468, βTC-3, and human fibroblast (HFb) cell lines with high release percent and without significant cytotoxicity. The DOX–CHS–GNPs and DOX–CHS–GNP-CS NPs showed a mean size of 175.8 ± 1.94 and 208.9 ± 2.08 nm; furthermore, a zeta potential of − 34 ± 5.6 and − 25.7 ± 5.9 mV, respectively. The highest release of DOX was 73.37% after 45 h, while in the absence of CS, the release of DOX was 76.05% for 24 h. Compared to CHS-GNPs, the presence of CS decreased the rate of sustained release of DOX and improved the drug release efficiency. The results demonstrated an excellent release and negligible cytotoxicity at high concentrations of CHS-GNP-CS. Consequently, in ovo assessment corroborated the efficacy of the green fabricated NPs proposed effective targeted delivery of DOX for anti-tumor therapy in vitro.

**Graphical Abstract:**

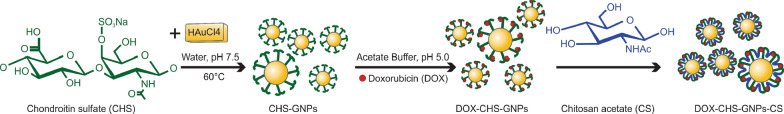

**Supplementary Information:**

The online version contains supplementary material available at 10.1186/s13065-022-00895-x.

## Introduction

Doxorubicin (DOX) is an efficient anthracycline chemotherapeutic for treating solid tumors, particularly desmoplastic cancers such as breast and pancreatic cancers. Two main pathways for the DOX mechanism of action are considered: intercalating with DNA leading to topoisomerase II inhibition and the generation of reactive oxygen species leading to plasma membrane damage [1–4]. Despite superb performance in cancer treatment, undesirable cytotoxicity reported on different tissue, including the heart and gut, has limited the systemic delivery of DOX. For instance, irreversible cardiomyopathy has been reported due to the cumulative drug dose and consequent cell damage resulting from the enhanced free radical in myocytes [5, 6].

Nanoparticles (NPs) have shown an exciting capacity for drug delivery systems (DDS) with adjustable and sustained profile release of drugs and devising platforms for targeted delivery with minimal side effects [7]. Many DDS types have been introduced through encapsulation or conjugation of different NPs for targeted delivery of DOX with low cytotoxicity and enhanced profile extended-release on tumor sites [8]. In addition to FDA-approved liposomal forms of DOX delivery [9], a wide variety of NPs have been formulated for systematized release of the drug, including liposome [10], inorganic NPs [11–13], polymeric NPs [14, 15], conjugates [14–17], micelles [18, 19], silica NPs [20] and exosomes [21, 22]. Gold nanoparticles (GNPs) have shown promising results for DDS purposes. Appropriate biocompatibility, tunable surface chemistry, capability to conjugate with different bioactive molecules, and adjustable particle size have turned this nanomaterial into a reliable candidate for designing novel tailored-made carriers for therapeutic and diagnostic applications [23–26]. Surface modification is an essential step in GNPs preparation to improve stability within an aqueous solution, minimize NPs aggregation, and restrain existing positive surface charges [27–29]. Importantly, detailed surface modification of GNPs is critical for tissue-specific delivery of solubilized formulations through the circulatory system while showing minimum toxicity [30–32].

Chondroitin sulfate (CHS) is a naturally occurring polysaccharide with repeating disaccharide units of b-1,4-linked d-glucuronic acid and b-1,3-linked N-acetyl galactosamine [33]. This glycosaminoglycan has been successfully employed to prevent the aggregation of GNPs. The polyanionic property of CHS reduces the positive charges of the gold atom, encloses GNPs’ surface, and subsequently forms an efficient capping agent for enhancing GNPs’ stability, biocompatibility, and solubility [34, 35]. Moreover, chitosan (CS) is accepted as a promising drug delivery vector due to the primary amino groups' presence on this biodegradable polysaccharide [36]. The bioadhesive properties of CS can prolong the retention time of NPs in the bloodstream, thus improve the cellular internalization of NPs and the release rate of pharmaceutics [37, 38]. Although the conjugation of GNPs with various ligand moieties for efficient delivery of Dox was successful, the efficient loading and sustained release of targeted GNPs for Dox delivery were unsolved. In a targeted case study, Aptamers (Apts)-CS-GNPs using nucleolin aptamers (AS1411) were developed for co-delivery Dox, and FOXM1 aptamer has efficiently enhanced mortality in 4T1 (breast) and A549 (lung) cancer cells [39]. A combination of Dox-loaded thiol-modified chitosan GNPs and photothermal therapy against cancer cells demonstrated good biocompatibility and low cytotoxicity of the construct [40]. Finally, an efficient pH-dependent targeted DOX delivery was utilized to cancer cells through immobilization of Dox on GNPs was capped with carboxymethyl chitosan (CMC). The system demonstrated pH-triggered drug release to overcome drug resistance in HeLa (Human cervical cancer) [41].

Moreover, GNPs functionalized by sodium 3-mercaptopropane sulfonate (GNP-3MPS) were synthesized to evaluate the release kinetics of dexamethasone (DXM) for inhibition of cell proliferation and apoptosis on a human lymphoma cell line, and upregulation of DXM-inducible programmed cell death-1 (PD-1) molecule on activated mouse T lymphocytes [42]. In another research study, a controlled-release anticancer therapy was assessed by GNPs with two different copper (I) complexes, including [Cu(PTA)_4_] + [BF_4_] (loading 90% and slow release) and [HB(pz)Cu(PCN)] (loading 10%) was demonstrated [43]. Comparing other mentioned targeted ones, the release profile of Dox with GNPs NPs not only must supply reproducible and flexible sustain release of Dox but also corroborates GNPs as a potential alternative for the functionalized carrier with at least burst release.

In this study, a novel and effective strategy for tumor-targeted DOX delivery is introduced based on the functionalization of GNPs with CHS and loading DOX molecules on this stable and nontoxic compound. The drug sustain release was achieved by adding CS as the delivery vector. Our approach in DOX delivery can minimize the occurrence of cardiac toxicity. The efficacy of the suggested green fabricated GNPs was evaluated by examining the response of three different cell lines, including MDA-MB-468 (human breast cancer cells), βTC-3 (mouse pancreatic β-cells), and human fibroblast (HFb) cell lines.

## Materials and methods

### Materials

Chondroitin 4-sulfate sodium salt from a bovine trachea, low molecular weight chitosan (75–85% DDA and 50–190 kDa), and chloroauric acid 99.99% (HAuCl_4_) were purchased from Sigma-Aldrich (St. Louis, MO, USA). 3-(4,5-Dimethylthiazol-2-yl)-2,5-diphenyltetrazolium bromide (MTT) was purchased from Roche (Mannheim, Germany). Doxorubicin HCl (DOX) was obtained from Actoverco pharmaceutical company (Tehran, Iran). Dulbecco’s Modified Eagle’s Medium (DMEM), fetal bovine serum (FBS), antibiotic solutions, and phosphate-buffered saline (PBS) were obtained from Gibco (NY, USA). All other materials were purchased from Merck (Darmstadt, Germany) and were analytical reagent grade. Chitosan was further purified by the precipitation procedure [44].

### Cell lines and culture

Briefly, MDA-MB-468 cells were cultured in DMEM supplemented with 10% FBS, 100 IU mL^−1^ penicillin, 100 IU mL^−1^ streptomycin at 37 °C in a humidified incubator with 5% CO_2_ levels. Then, the growth patterns and morphology of cells were regularly examined using an inverted microscope [45]. After 48 h incubation, the cells were harvested by trypsinization to perform cell passaging and backup cultures. Furthermore, βTC3 cells in the presence of 2 mM l-glutamine were cultured similarly in a proper culture medium [45, 46]. Human fibroblast as the normal cell was isolated by the procedure described in our previous work [45, 47]. All the cell culture ingredients were supplied from Auto-cell Co. (Warsaw, Poland).

### Synthesis and preparation of NPs

#### Green synthesis of GNPs capped with CHS (CHS-GNPs)

20 ml of HAuCl_4_ (0.39 mg mL^−1^) with 10 mL of CHS (5 mg mL^−1^) were reacted (in a 2:1 ratio v/v %) while the pH of the solution was maintained at 7.5 by NaOH solution (0.1 N) and HCl solution (0.1 N). The solution was stirred at 60 °C for 70 min until a deep-red color appeared. After three times centrifuging (8000 rpm, 30 min), the reaction was performed in a stirring aqueous environment at room temperature without the need for UV light, autoclave, microwave, and laser irradiation (CHS-GNPs) [34, 48].

#### Conjugation of DOX and CHS-GNPs

The conjugation of DOX onto CHS-GNPs was performed by mixing 2 mg of CHS-GNPs with 20 µL DOX (1% w/v) in distilled water with a ratio of 1:10 w/w. The product was shaken at 37 °C and 100 rpm for 30 min. It is assumed that positively charged DOX can be complexed with negatively charged CHS-coated GNPs through electrostatic interaction. Eventually, the solution was centrifuged at 14,000 rpm for 20 min. The yielded pellet was dispersed in 300 μL of distilled water to determine the percentage of CHS-GNPs loaded with DOX.

#### Modification of DOX-CHS-GNPs by CS

For this purpose, 3 µL of CS (2 w/v %**)** was added dropwise to 300 µL of DOX-CHS-GNPs solution (in a ratio of 1:100 v/v %). Then, the mixture was shaken for 3 min, incubated at room temperature for 30 min, and centrifuged at 14,000 rpm for 20 min. The DOX loading and supernatant concentrations were determined. Different DOX concentrations (3.125, 6.25, 12.5, 25, 60, 80, and 100 μgmL^−1^) in distilled water were prepared to evaluate the amount of drug-loaded on the surface of synthesized NPs. The DOX concentration in the supernatant and the precipitate was determined using UV–Vis spectroscopy at 480 nm. A step-by-step schematic procedure for preparing DOX-CHS-GNPs-CS by the green synthesis method was presented in Additional file 1: Fig. S1.

### Physicochemical properties of NPs

UV–Vis spectroscopy Cary 100 (Varian) is a reliable technique to evaluate the formation, and crystal growth of NPs. [48]. To this end, the solution of DOX-CHS-GNPs-CS with Dox concentration 20 μg mL^−1^ was diluted by distilled water in a 2:1 ratio v/v % at pH 7 and prepared using UV–vis spectrophotometry.

To verify the DOX-CHS-GNPs-CS synthesis, FT-IR spectra were recorded by Prestige-21 Shimadzu Spectrometer (Kyoto, Japan). The suitable amount of samples, including CHS, CHS-GNPs, DOX, DOX-CHS-GNPs, CS, and DOX-CHS-GNPs-CS, was compressed into KBr, and samples were then tested in the range of 400–4000 cm^−1^ with a resolution of 4 cm^−1^. Furthermore, particle size, zetapotential and polydispersity index of CHS-GNPs, DOX-CHS-GNPs, and DOX-CHS-GNPs-CS were evaluated using DLS following distilled water in a 1:9 ratio v/v % at pH 7.

Transmission electron microscopy (TEM) was used to identify the morphology and particle size of CHS-GNPs and DOX-CHS-GNPs by using LEO 906 E microscope (Carl Zeiss, Germany) with an accelerating voltage of 100 kV. Before analysis, GNPs were deposited on carbon-copper grids. In addition, the surface morphology of DOX-CHS-GNPs-CS was investigated through TEM. Finally, GNPs represent consistent stability between − 30 and + 30 mV of their surface potential values because ambient ionic liquids can produce electrostatic forces to increase GNPs stability which was quite evident from higher zeta potential value versus aqueous extract value [49, 50].

### X-ray diffraction (XRD) analysis

To confirm the crystalline nature of the synthesized CHS-GNPs, XRD analysis was carried out (Xpert pro, Panalytical, Holland), which was operated at a voltage of 40 kV and a current of 30 mA with cu k^−1^ radiation.

### In vitro cytotoxicity assay

The MTT assay was utilized to evaluate the cytotoxicity of free DOX and NPs-DOX on MDA-MB-468, βTC-3, and HFb cell lines, as described previously [46, 51]. To evaluate cell viability, 1 × 10^4^ cells were cultured in a 24-well plate with the proper culture medium for 24 h. Then, 20 µL of treatment solutions of DOX, DOX-CHS-GNPs, and DOX-CHS-GNPs-CS, were added at different concentrations into each well for another 48 h. Finally, the cell viability was evaluated by analyzing optical densities (ODs) obtained from the SpectraMax 190 absorbance microplate reader (Bio Tek Instruments, USA) at 570 nm.

### In vitro release study of DOX

In order to evaluate the release of DOX at the surface of DOX-CHS-GNPs and DOX-CHS-GNPs-CS, cellulose acetate dialysis membranes (Spectra, MWCO 12 kDa) in phosphorus buffer solution (PBS) at pH 7.4 were used [46, 52]. DOX-CHS-GNPs and DOX-CHS-GNPs-CS were located inside the membrane separately and were dialyzed against 7 mL PBS for continuous stirring at 100 rpm. Every hour, to read the absorbance of DOX, a total solution of 7 mL of PBS dialysis solution was replaced completely with 7 mL of fresh PBS. The amount of released DOX was analyzed by determining the absorbance at 481 nm, and the release of DOX was calculated according to the standard equation of the released drug. The released DOX amounts from DOX-CHS-GNPs and DOX-CHS-GNPs-CS were measured in the different time intervals (0.25, 0.5, 1, 2, 3, 4, 5, 6, 18, 24, 27, 30, 42, and 45 h). Finally, the results were repeated three times for each sample, and the calibration curve of DOX in the incubation medium was calculated.

### CAM assay

Chick chorioallantoic membrane (CAM) assay was used to evaluate the effect of different therapeutic groups on reducing angiogenesis and tumor growth. In summary, fertilized hen’s eggs were incubated at 37 °C and 60% humidity. After 10 days, an incision (square dimension: 0.5 cm^2^) was made on the eggshell under sterile conditions to access the CAM layer. Subsequently, 0.5–1 × 10^6^ cells (10 µl of the cell suspension) treated with DOX-CHS-GNPs-CS, DOX-CHS-GNPs, free DOX, and CS-CHS-GNPs were injected onto the CAM surface. The incised area was then covered, and the eggs were reincubated for 7 days. Finally, the shell was opened, and CAM was extracted to assess tumor weight, size, and angiogenesis, and images were taken.

## Results and discussion

### Structural and physicochemical analyses

#### FT-IR assessment

**Fig. 1:**
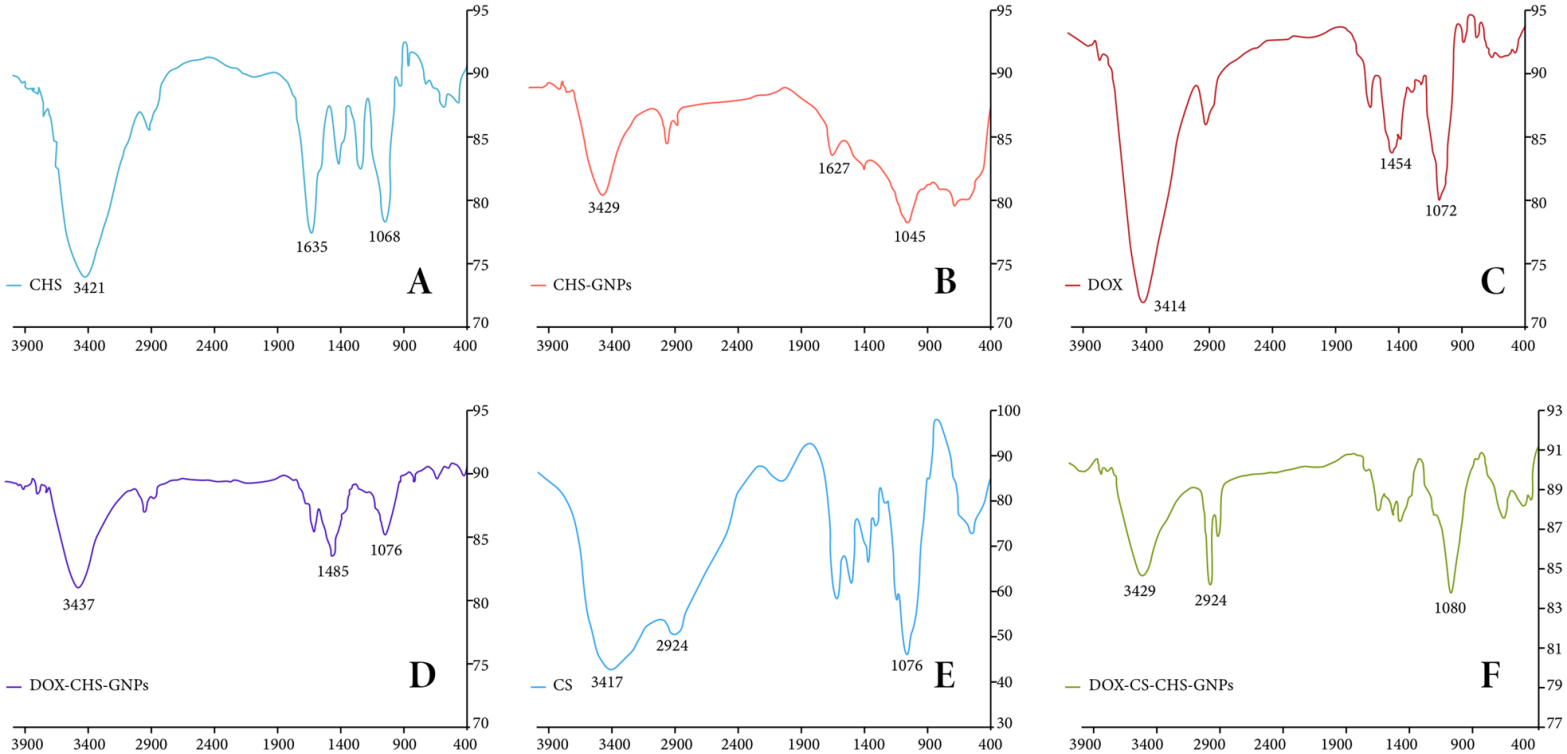
FT-IR spectrum of CHS (**A**), CHS-GNPs (**B**), DOX (**C**), DOX-CHS-GNPs (**D**), CS (**E**), and DOX-CHS-GNPs-CS (**F**)

As shown in Fig. 1A, the FT-IR spectrum of CHS depicts the broad peaks in the region around 2600–3650 cm^−1^ and 2924 cm^−1^, which indicates the stretching vibration of hydroxyl groups (OH) and CH_2_ and CH_3_ asymmetric stretching vibration aliphatic. Furthermore, the peaks at 1190 and 1064 cm^−1^ show two C–O stretching vibrations of O–H, and the other peaks at 3425 and 1566 cm^−1^ indicate N–H stretching vibration and bending vibration of the secondary amine of CHS, respectively [53, 54]. Subsequently, the peaks at 1242 cm^−1^ and 1651 cm^−1^ show N–C stretching vibration and C=O, the carboxylic acid carbonyl stretching vibration. As shown in Fig. 1B, the FT-IR spectrum of CHS-GNPs confirmed the wavelength decreasing of the C=O group from 1651 to 1694 cm^−1^ due to the presence of gold molecules, the carboxylic acid group of CHS has reduced to the ketone [48].

The FT-IR spectrum of DOX (Fig. 1C) depicts a broad peak at 2700–3650 cm^−1^ for -OH stretching vibration and two peaks at 3414 and 1624 cm^−1^ for N–H stretching vibration and bending vibration of the primary amine, respectively. Furthermore, the peaks at 1215 and 1072 cm^−1^ indicate to C–O stretching vibration of DOX, and the peaks at 1577 and 1454 cm^−1^ show C=C stretching vibrations of the aromatic ring.

The FT-IR spectrum of DOX-CHS-GNPs (Fig. 1D) depicts the peaks at 3437, 1627, and 1583 cm^−1^ that are assigned to N–H stretching vibration, and bending vibration of the primary and secondary amine of DOX-CHS, respectively. In addition, the peak at 1689 cm^−1^ shows C=O stretching vibrations of DOX and CHS units from DOX-CHS-GNPs. Finally, the shifts in the peak positions from 3425 and 3414 to 3437 cm^−1^ and also 1651 and 1708 to 1689 cm^−1^ confirmed the presence of DOX and CHS in the spectrum of DOX-CHS-GNPs. As shown in Fig. 1D, the intensity of the peaks in the spectrum of DOX-CHS-GNPs was lower than DOX and CHS peaks. The FT-IR spectrum of CS (Fig. 1E) depicts the peaks at 2924 and 2893 cm^−1^ related to C–H asymmetric stretching vibrations. The peak at 1523 cm^−1^ indicates the bending vibration of the primary amine. The peak at 1072 cm^−1^ shows the C–O stretching vibration of CS [48, 55]. The FT-IR spectrum of DOX-CHS-GNPs-CS (Fig. 1F) depicts the sharp peaks at 3429, 1627, and 1570 cm^−1^ representing the N–H stretching vibration of DOX and bending vibration of the primary amine of CS and secondary amine of CHS, respectively. These results confirmed the presence of DOX, CS, and CHS in the spectrum of synthesized NPs.

#### XRD analysis

**Fig. 2:**
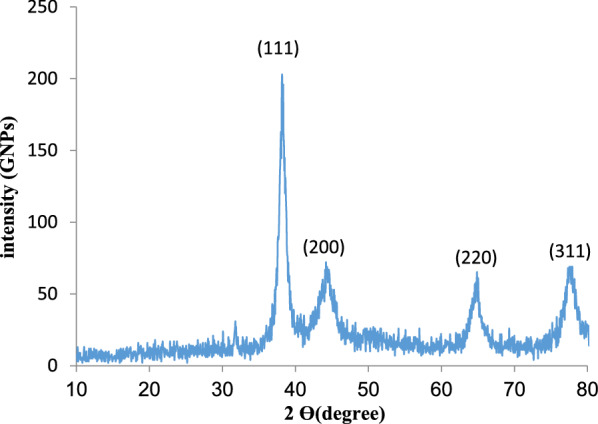
XRD spectrum of synthesized GNPs

The XRD analysis recognizes the purity and crystal structure of the central construct of synthesized NPs. Figure 2 shows the XRD pattern of the synthesized CHS-GNPs, which revealed four well-defined characteristic peaks in the 2θ range (10–80°). The diffraction peaks of CHS-GNPs were detected at 37.8°, 44.6°, 64.6°, and 77.5°, which corresponded to (111), (200), (220), and (311) crystalline planes, respectively. This presented a reflection of the face center cubic structure of CHS-GNPs, showing that the synthesized GNPs are composed of crystalline. The XRD pattern showed no additional peak, which refers to the high purity of CHS-GNPs.

#### NPs characterization

**Fig. 3:**
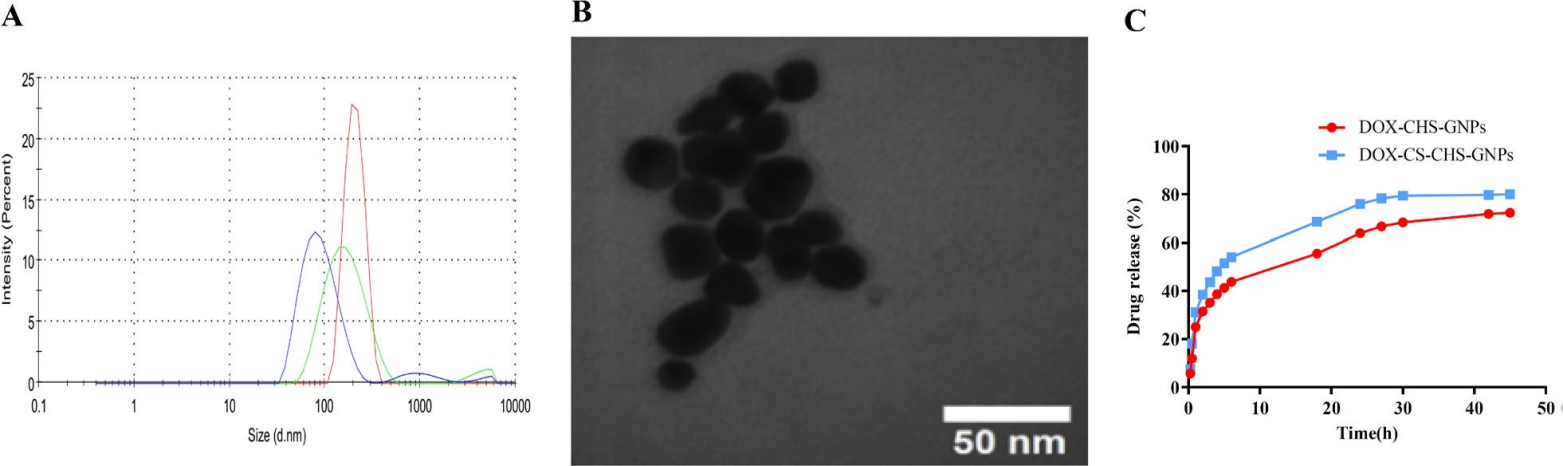
**A** Particle size distribution of CHS-GNPs (Blue line), DOX-CHS-GNPs (Green line), and DOX-CHS-GNPs-CS (Red line), **B** TEM image of DOX-CHS-GNPs, and **C** drug release of free DOX from DOX-CHS-GNPs and DOX-CHS-GNPs-CS in PBS (pH 7.4)

Following the successful synthesis and preparation of DOX–CHS–GNP–CS, DLS was used to evaluate of NPs characterization. To that end, the DLS illustrates the average hydrodynamic diameter (Dh) of CHS-GNPs and DOX-CHS-GNPs 93.75 ± 2.52 nm and 175.8 ± 1.94 nm, respectively (Fig. 3A). Furthermore, Fig. 3B shows the TEM image of the synthetic NPs, which demonstrates an entirely dense and homogeneous morphology.

As shown in Table 1, the average size of DOX-CHS-GNPs is larger than CHS-GNPs due to the swelling of DOX around CHS-GNPs. PDI of CHS-GNPs and DOX-CHS-GNPs is 0.241 ± 0.06 and 0.244 ± 0.08, which demonstrate highly polydisperse [56]. The surface charge or zeta potential (stability) of DOX-CHS-GNPs and DOX-CHS-GNPs-CS were − 34 ± 5.7 and − 25.70 ± 5.90 mv, respectively, which confirms their proper stability. The presence of negative charges on CHS minimized the accumulation of CHS-GNPs, leading to stabilized NPs dispersion. As seen in Fig. 3A, B, the diameter of CHS-GNPs was larger after DOX loading, indicating successful DOX loading. It is noteworthy that the size of NPs, measured by the TEM, is different from what the DLS reported due to the differences in the way they work [57].

**Table 1:** Physicochemical characteristics of CHS-GNPs, DOX-CHS-GNPs, and DOX-CHS-GNPs-CS

Formula	Size (nm) (DLS)	Size (nm)(TEM)	Zeta potential (mV)	PDI	Yield (%)	DOX loading (%)
CHS-GNPs	93.75 ± 2.52	83 ± 5.0	− 15.50 ± 5.09	0.241 ± 0.06	86.11 ± 2.0	–
DOX-CHS-GNPs	175.8 ± 1.94	147 ± 5.0	− 34.00 ± 5.67	0.244 ± 0.08	89.73 ± 5.0	89.10 ± 3.02
DOX-CHS-GNPs-CS	208.9 ± 2.08	191 ± 5.0	− 25.70 ± 5.90	0.12 ± 0.03	–	84.07 ± 1.15

DLS shows the particles' hydrodynamic diameter (Dh), including the inorganic core and any coating developed around the core, such as the hydration layer. On the other hand, TEM estimates the projected area pore diameter (Pd), which is the equivalent hard-core diameter of the particle [58]. Hence, the particle size from the DLS technique is larger than TEM due to the presence o the CHS compound around the core of GNPs. Moreover, the surface morphology of CS-CHS-GNPs with loaded DOX was investigated.

#### Drug loading and release studies

The amount of drug loading was tested through the mass-ratio changes of 10, 20, and 30%; therefore, the optimized mass ratio of 10% was selected. At this ratio, the mass percentage of the drug was loaded about 89%. Moreover, the ability to selectively release drugs demonstrates the optimal synthesis route for preparation with the well-defined characterization of the NPs [46, 47]. The release studies were performed under ambient conditions for the first 6 h of treatment with NPs. As shown in Fig. 3C, the release of DOX amounts from DOX-CHS-GNPs and DOX-CHS-GNPs-CS in PBS was illustrated. In the first 6 h of treatment with NPs, 54% of DOX from DOX-CHS-GNPs was released, while at the end of 24 h, the release reached 76.05%. However, the release of the drug from DOX-CHS-GNPs-CS was slower than DOX-CHS-GNPs due to the presence of CS, at the end of the first 6 h of treatment with NPs, 43.8% of DOX was released from DOX-CHS-GNPs-CS. The highest DOX release from DOX-CHS-GNPs-CS was indicated at 73.37% after 48 h, while in the absence of CS, the DOX release was 76.05% for 24 h. Due to the presence of CS at the surface of DOX-CHS-GNPs, the process of releasing DOX was prolonged over time, which improved efficient drug release.

### Cell viability assay

**Fig. 4:**
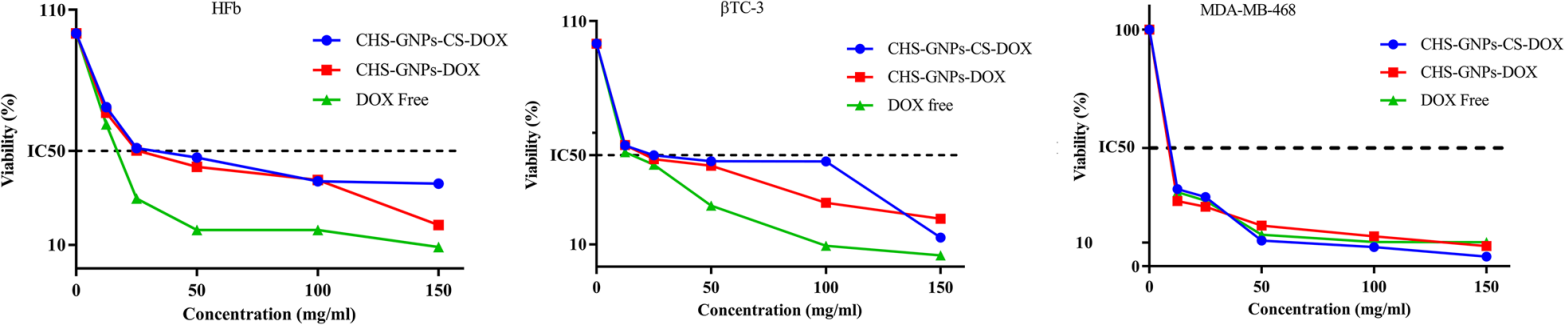
In vitro cytotoxic of DOX, DOX-CHS-GNPs, and DOX-CHS-GNPs-CS against human fibroblast, MDA-MB-468, and βTC-3 cells at various concentrations after 48 h. IC_50_ value was measured by plotting the log10 percentage of proliferation values versus DOX concentrations

MDA-MB-468 and βTC-3, and HFb cells were exposed to free DOX, DOX-CHS-GNPs, and DOX-CHS-GNPs-CS at different concentrations of 12.5, 25, 50, 100, and 150 μg mL^−1^ over 48 h (Fig. 4). Furthermore, a fluorescence microscopy comparison of HFb cells, MDA-MB-468, and βTC-3 cells with DOX-CHS-GNPs-CS treatment was shown in Additional file 1: Fig. S2.

Fibroblasts exhibited the highest viability after exposure to DOX-CHS-GNPs-CS compared to two cancer cell lines, which means less toxicity of DOX-NPs on the normal cells. MDA-MB-468 and βTC-3 cells showed the lowest viability in exposure to DOX-CHS-GNPs-CS compared to DOX-CHS-GNPs. On the other hand, DOX-CHS-GNPs-CS had the least effect on normal cells than cancer cells, proving targeted therapy with fewer side effects. The toxicity of DOX-CHS-GNPs-CS in normal fibroblasts significantly increased at concentrations above 12.5 μg mL^−1^_,_ and thus, the viability rate was reduced. DOX-CHS-GNPs-CS showed a lower effect on the βTC-3 compared to MDA-MB-468 cells. The findings indicate that βTC-3 cells are less responsive to DOX chemotherapy. It can be concluded that the low internalization of NPs by this cell line resulted in a milder response of the cells in comparison with treated cells with free DOX. The comparison of IC_50_ values is summarized in Table 2.

**Table 2:** IC_50_ values following treatment of free DOX, DOX-CHS-GNPs, and DOX-CHS-GNPs-CS in the cell lines

Formula	IC_50_ (µg mL^−1^)		
	MDA-MD-468	βTC	Fibroblast
Free DOX	< 12.5	= 12.5	> 12.5
DOX-CHS-GNPs	< 12.5	= 12.5	= 12.5
DOX-CHS-GNPs-CS	< 12.5	> 12.5	> 12.5

IC_50_; The half-maximal inhibitory concentration

The difference in the effect of DOX-CHS-GNPs-CS on normal cells and tumor cells could be related to CHS interaction with CD44 receptors in the cancer cell membrane. Although CD44 is widely expressed on various cell lines, the level of distribution, variety in isoforms, and nature of cell lines explain the differences in MTT results. Our results comply with previous studies showing MDA-MB-468 cells are more susceptible to CHS-GNPs mainly because of CD44-high expression [59–61].

### The effect of DOX-CHS-GNPs-CS on angiogenesis of studied cells

**Fig. 5:**
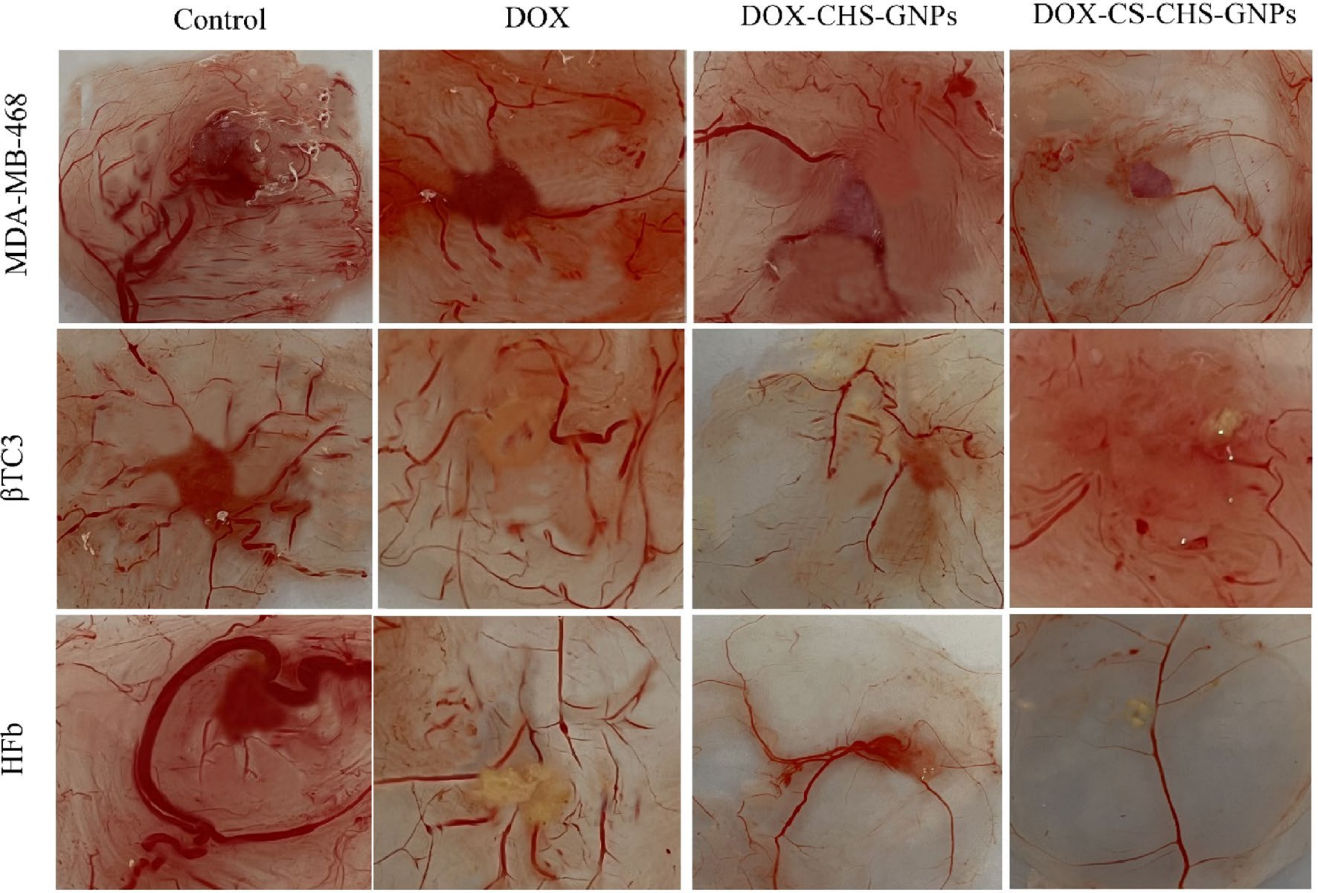
CAM assay was utilized to investigate the effect of combination therapy on the angiogenesis rate as well as the size and weight of the tumors (**A**–**C**)

**Fig. 6:**
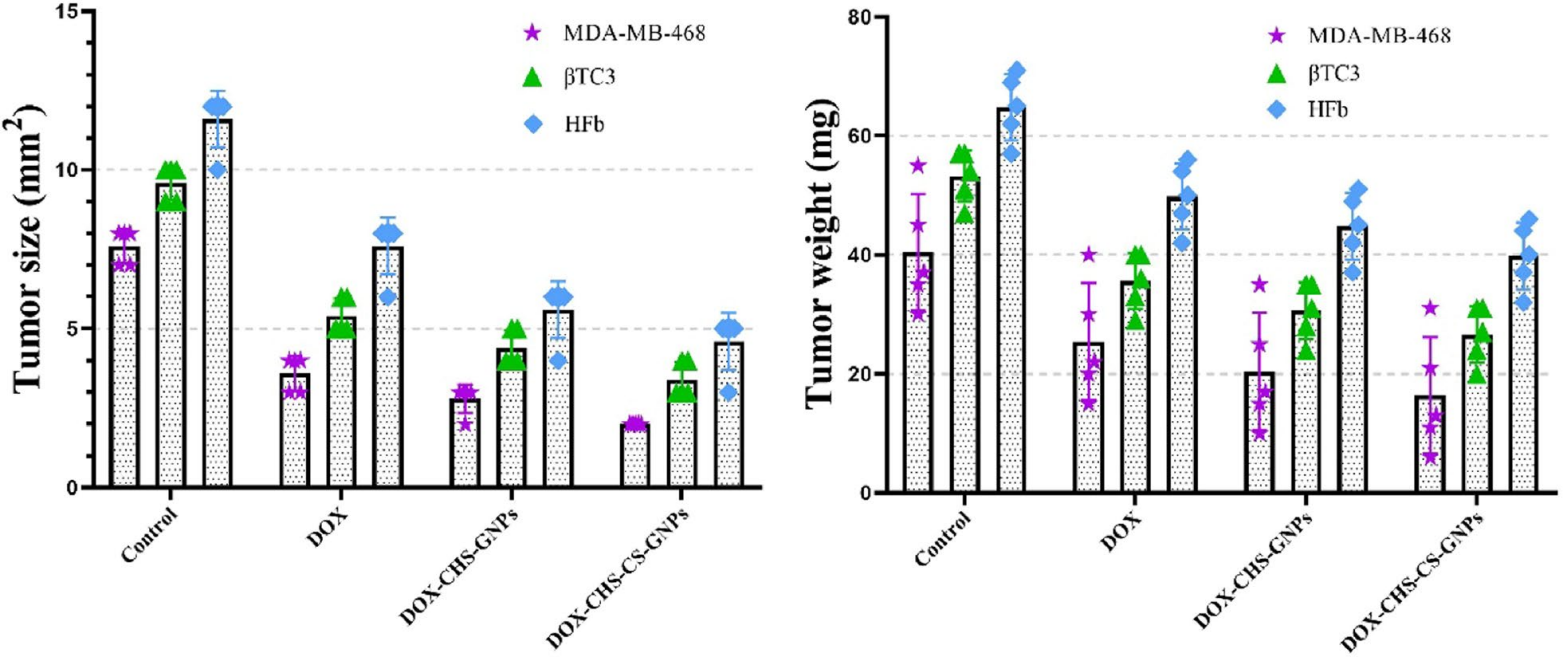
The tumor size (left) and tumor weight (right) of CHS-GNPs, DOX-CHS-GNPs, and DOX-CHS-GNPs-CS for release of DOX

The CAM assay was used to evaluate the potential of DOX-encapsulated CS-CHS-GNPs in suppressing angiogenesis and reducing tumor size. As illustrated in Fig. 5, the angiogenesis rate in cells treated with DOX-CHS-GNPs-CS and DOX-CHS-GNPs (compared with free DOX and control group) was significantly reduced. Besides, treatment with DOX-loaded CS-CHS-GNPs dramatically reduced the size and weight of tumors formed on the CAM layer (Fig. 6). According to the evidence, the impact of pharmaceutic groups on the growth and angiogenesis of MDA-MB-468 cells is more significant than βTC3 and normal cells, respectively, which may be due to the higher cellular uptake of NPs by these cells.

### Statistical analysis

All experiments were assessed in triplicate wells and repeated twice, and data have been shown as mean ± standard error for at least three independent samples. Data were analyzed by using the student’s t-test with SPSS software (version 17.0). The statistical comparison between groups and within groups was performed using one-way ANOVA (*p-values < 0.05).

## Conclusion

Development of efficient delivery requires sustained drug release at a programmed rate which remains a prolonged period for the precise release of the drug. Manipulation of various biocompatible nanomaterial facilities the controlled and extended-release, likewise selection of CS as a biodegradable, regularly shaped polyhedron and semicrystallisation polymer demonstrated controlled release of the DDS. The presence of CHS, as an excellent stabilizing agent, increases the stability of CHS-GNPs. The increase in size and zeta potential with keeping PDI exhibited controllable release and optimized preparation of NPs for Dox delivery. Additionally, the cytotoxicity activity of DOX-CHS-GNPs-CS on MDA-MB-468, βTC-3, and HFb cells demonstrates that the construct promise a safe, sustained release profile and efficient DDS for soluble in water chemotherapy drugs. Considering these significant features, the green synthesized CS-CHS-GNPs hold tremendous potential for DOX delivery to the cancer cells with a lower damage effect on normal cells. Subsequently, in ovo studies corroborated that treating malignant cells with DOX-CHS-GNPs-CS could significantly inhibit tumor growth and angiogenesis to mimic an in vivo model. Taken together, the findings suggest GNPs as an effective drug delivery system for the treatment of breast and pancreatic cancers that should be further evaluated in the future.

## Supplementary Information

**Additional file 1: Figure S1.** The step-by-step schematic procedure for synthesized process of DOX-CS-CHS-GNPs. **Figure S2.** Fluorescence microscopy comparison of human fibroblast, MDA-MB-468, and βTC-3 with DOX-CS-CHS-GNPs treatment at various concentrations after 48 h.

## Data Availability

The datasets generated and/or analyzed during the current study are not publicly available due feasibility of the study to begin a clinical trial; however, they are available from the corresponding author upon reasonable request.
