# Supplementary material for: Extended-release of doxorubicin through green surface modification of gold nanoparticles: in vitro and in ovo assessment
Source: BMC Chem. 2022 Dec 6;16(1):110. doi: 10.1186/s13065-022-00895-x (PMC9724295; doi:10.1186/s13065-022-00895-x)
Supplement: Supplementary file 1 — Additional file 1: Figure S1. The step-by-step schematic procedure for synthesized process of DOX-CS-CHS-GNPs. Figure S2. Fluorescence microscopy comparison of human fibroblast, MDA-MB-468, and βTC-3 with DOX-CS-CHS-GNPs treatment at various concentrations after 48 h. [file 13065_2022_895_MOESM1_ESM.docx]

**Supplementary Materials**

**Extended-Release of Doxorubicin through Green Surface Modification of Gold Nanoparticles: *in Vitro* and *in Ovo* Assessment**


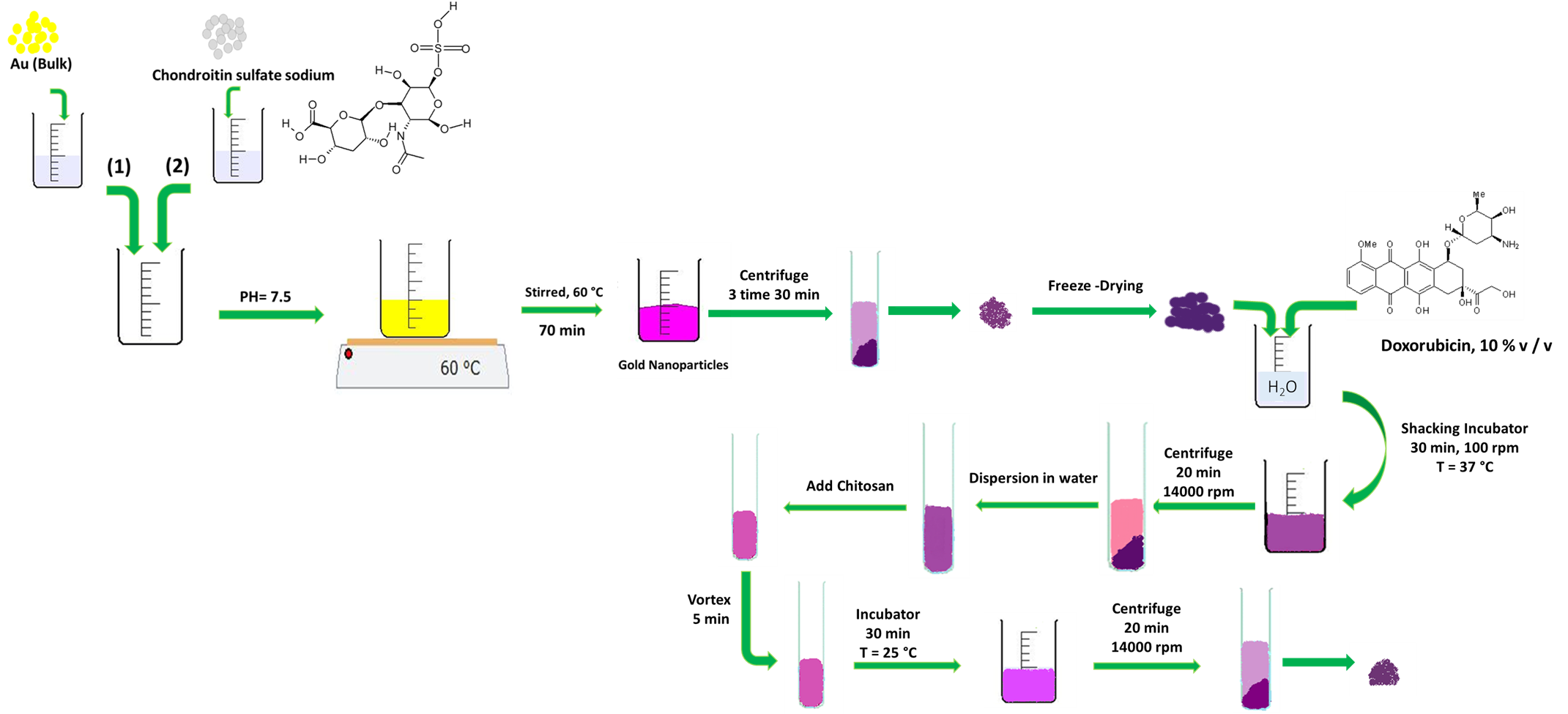


**Figure S1.** The step-by-step schematic procedure for synthesized process of DOX-CS-CHS-GNPs.

|  | Human Fibroblast | MDA-MD-468 | βTC-3 |
| --- | --- | --- | --- |
| Control | 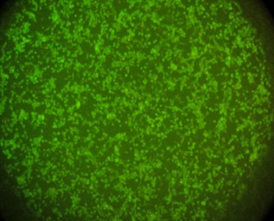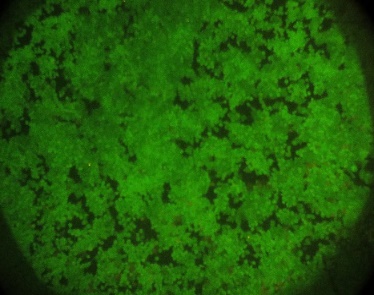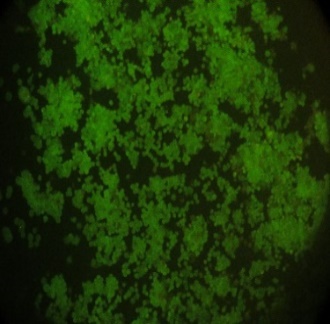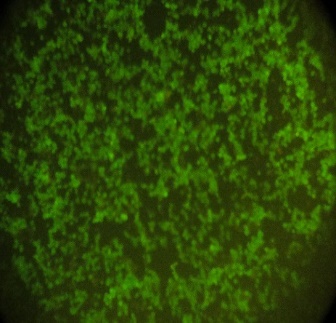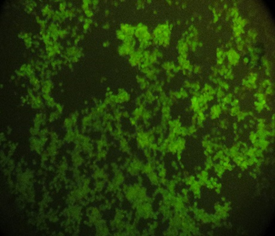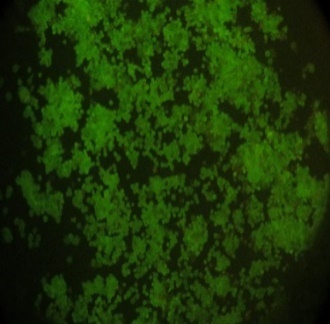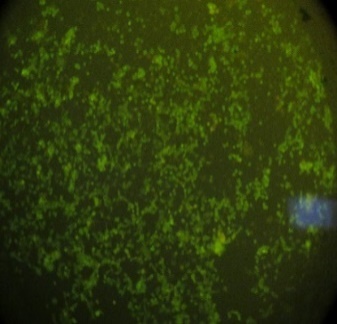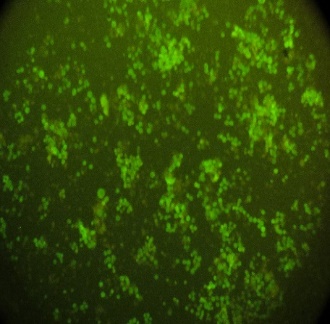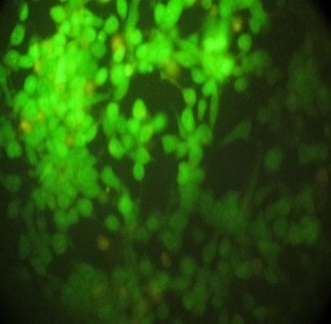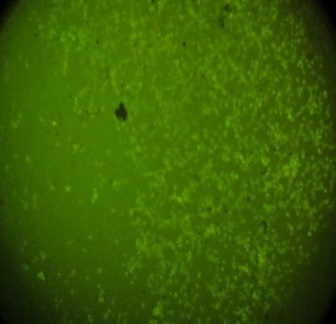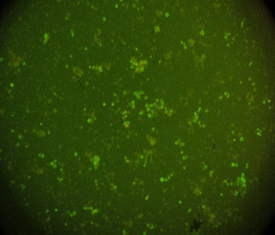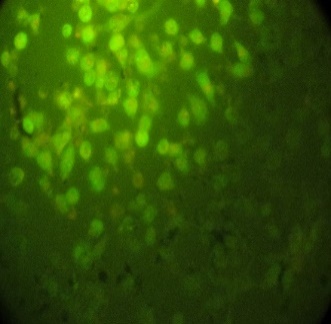 | | |
| 250(µgml^-1^) |  |  |  |
| 500(µgml^-1^) |  |  |  |
| 1000(µgml^-1^) |  |  |  |

**Figure S 2.** Fluorescence microscopy comparison of human fibroblast, MDA-MB-468, and βTC-3 with DOX-CS-CHS-GNPs treatment at various concentrations after 48 h.
